# Identification of Makorin 1 as a novel SEREX antigen of esophageal squamous cell carcinoma

**DOI:** 10.1186/1471-2407-9-232

**Published:** 2009-07-15

**Authors:** Hideaki Shimada, Tooru Shiratori, Mari Yasuraoka, Akiko Kagaya, Mari Kuboshima, Fumio Nomura, Masaki Takiguchi, Takenori Ochiai, Hisahiro Matsubara, Takaki Hiwasa

**Affiliations:** 1 Department of Gastroenterological Surgery, Chiba Cancer Center, Chiba, Japan; 2 Department of Frontier Surgery, Chiba University, Graduate School of Medicine, Chiba, Japan; 3 Department of Biochemistry and Genetics, Chiba University, Graduate School of Medicine, Chiba, Japan; 4 Department of Molecular Diagnosis, Chiba University, Graduate School of Medicine, Chiba, Japan; 5 Gastroenterological Center, San-Ai Memorial Hospital, Chiba, Japan

## Abstract

**Background:**

Esophageal squamous cell carcinoma (SCC) represents one of the most malignant tumors. To improve the poor prognosis, it is necessary to diagnose esophageal SCC at early stages using new tumor markers. SEREX (serological identification of antigens by recombinant cDNA expression cloning) is suitable for large-scale screening of tumor antigens and has been applied for various types of human tumors.

**Methods:**

Tumor markers of esophageal squamous cell carcinoma (SCC) were screened by SEREX method. The presence of serum anti-makorin 1 (MKRN1) antibodies (s-MKRN1-Abs) was examined by Western blotting using bacterially expressed MKRN1 protein. The expression levels of MKRN1 mRNA in tissues were examined by RT-PCR. The biological activity of MKRN1 was examined by transfection of ras-NIH3T3 mouse fibroblasts with MKRN1 cDNA. Major ubiquitinated proteins in MKRN1-transfected cells were identified by immunoprecipitation with anti-ubiquitin antibody followed by mass spectrometry.

**Results:**

MKRN1 was identified as a novel SEREX antigen of esophageal SCC. Although a total of 18 (25%) of 73 patients with esophageal SCC had s-MKRN1-Abs, none of the 43 healthy donors had a detectable level of s-MKRN1-Abs. There was no correlation between the presence of s-MKRN1-Abs and clinicopathological variables other than histological grading. Well-differentiated tumors were associated significantly with the presence of s-MKRN1-Abs in the patients. The mRNA levels of MKRN1 were frequently higher in esophageal SCC tissues than in the peripheral normal esophageal mucosa. Stable transfection of ras-NIH3T3 cells with MKRN1 cDNA induced prominent morphological changes such as enlargement of the cell body and spreading. Ubiquitination of 80- and 82-kDa proteins were clearly observed in MKRN1-transfected cells but not in the parental cells, which were identified as L-FILIP (filamin A interacting protein 1).

**Conclusion:**

MKRN1 is a novel SEREX antigen of esophageal SCC, and s-NKRN1-Abs can be a candidate of diagnostic markers of esophageal SCC with high specificity. It is plausible that MKRN1 is involved in carcinogenesis of the well-differentiated type of tumors possibly via ubiquitination of L-FILIP.

## Background

Esophageal squamous cell carcinoma (SCC) represents one of the most malignant tumors [1,2]. The poor prognosis of this tumor is attributed largely to a delay in diagnosis. Several tumor markers have been used for the diagnosis of esophageal SCC [3-5]. However, to improve the detection of esophageal SCC, new tumor markers are necessary. It has been known for several decades that the immune system is able to recognize tumor cells [6,7]. This was developed by Sahin *et al*. to establish a new method called SEREX (serological identification of antigens by recombinant cDNA expression cloning), which involves the immunoscreening of cDNA libraries prepared from tumor specimens with autologous or allogeneic sera [8]. Since clones isolated by SEREX can be easily identified by nucleotide sequencing, SEREX is suitable for large-scale screening of tumor antigens. SEREX screening of several different human tumor types has identified many novel tumor antigens [9,10].

A previous application of the SEREX method to esophageal cancer resulted in the identification of NY-ESO-1, a cancer-testis antigen expressed in various cancer cells but not in normal tissues [8]. SEREX analysis has also led to the isolation of several antigens with known cancer relatedness, including a mutated version of the p53 tumor suppressor protein, while the presence of antibodies to p53 in serum was associated with poor prognosis in esophageal cancer [11-13]. We applied the SEREX methodology to esophageal SCC with the aim of adding to the catalogue of defined immunogenic proteins in human cancer [14-21]. In this study, we report that makorin1 (MKRN1) is a new tumor antigen of esophageal SCC.

## Methods

### Human esophageal carcinoma cDNA library

This study was approved by the Ethic Committee of Chiba University, Graduate School of Medicine. Collection of tissues and sera was agreed upon by patients and healthy donors, after provision of written, informed consent. Recombinant DNA work was done with official permission and in accordance with the rules of the government of Japan. The human esophageal SCC cell line, T.Tn, was established in the Department of Clinical Molecular Biology, Chiba University, Graduate School of Medicine [22,23]. Total RNA was prepared from T.Tn cells by the acid guanidinium thiocyanate-phenol-chloroform method [24] and purified to poly(A)^+^RNA using Oligotex-dT_30 _(Super) mRNA Purification Kit (Takara Biochemicals, Kyoto, Japan) according to the manufacturer's instructions. cDNA was ligated into EcoRI-XhoI site of the λZAP II phage. The original library size was 1.8 × 10^6^.

### Sera of patients and healthy donors

Sera were obtained from 73 esophageal cancer patients with various stages of disease progression, as well as from 16 breast, 16 gastric, and 16 colorectal cancer patients prior to treatment. The patients with esophageal carcinoma consisted of 66 men (82%) and 7 women (18%), with a median age of 65 years (range 39 to 86 years). No treatment-related death was observed in these patients. They were pathologically classified according to the TNM/UICC classification [25] using resected specimens. The patients included 14 with stage I cancer, 21 with stage II, 27 with stage III, and 11 with stage IV cancer (Table 1). All patients underwent R0 resection with extended lymphadenectomy. After surgery, they were followed until the end of March 2006 or their deaths with clinical examinations and imaging studies on a regular basis. The median follow-up for survivors was 30 months. Each sample was centrifuged at 3,000 *g *for 5 min and then frozen at -80°C until use. Repeated thawing and freezing of samples was avoided.

**Table 1:** Relationship between s-MKRN1-Abs and the clinicopathological variables in 73 patients with esophageal SCC

		s-MKRN1-Abs		
			
		Positive	Negative	
		(18 patients)	(55 patients)	
Characteristics		n (%)	n (%)	P value
Age				
Median	65 yrs			
Range	39–86 yrs			
	
	Under 59 yrs	6 (33)	21 (38)	
	Over 60 yrs	12 (67)	34 (62)	0.711

Sex	Male	16 (89)	50 (91)	
	Female	2 (11)	5 (9)	0.555

Location	Ce	0 (0)	4 (7)	
	Ut	4 (22)	7 (13)	
	Mt	9 (50)	29 (53)	
	Lt	4 (22)	9 (16)	
	Ae	1 (6)	4 (7)	
	Unknown	0 (0)	2 (4)	0.682

Tumor size				
Median	58.6 mm			
Range	15–140 mm			
	
	Under 29 mm	2 (11)	8 (15)	
	Over 30 mm	13 (72)	47 (85)	
	Unknown	3 (17)	0 (0)	0.636

Histopathological Grading	GX	8 (44)	20 (37)	
	G1	6 (33)	5 (9)	
	G2	4 (22)	21 (38)	
	G3	0 (0)	9 (16)	0.008

Primary Tumor	Tis	0 (0)	1 (2)	
	T1	3 (17)	16 (29)	
	T2	4 (22)	10 (18)	
	T3	5 (28)	13 (24)	
	T4	6 (33)	15 (27)	0.824

Regional Lymph Nodes	N0	3 (17)	17 (31)	
	N1	15 (83)	38 (69)	0.194

Distant Metastasis	M0	16 (89)	46 (84)	
	M1	2 (11)	9 (16)	0.455

Stage Grouping	Stage 0	0 (0)	1 (2)	
	Stage I	1 (6)	12 (22)	
	Stage IIA	1 (6)	3 (5)	
	Stage IIB	6 (33)	11 (20)	
	Stage III	8 (44)	19 (35)	
	Stage IV	2 (11)	9 (16)	0.552

Tumor Markers				
SCC-Ag	Positive	7 (39)	19 (35)	
	Negative	11 (61)	32 (58)	
	ND	0 (0)	4 (7)	0.902

CYFRA21-1	Positive	3 (17)	13 (24)	
	Negative	15 (83)	38 (69)	
	ND	0 (0)	4 (7)	0.340

CEA	Positive	2 (11)	12 (22)	
	Negative	16 (89)	37 (67)	
	ND	0 (0)	6 (11)	0.199

### CEA, CYFRA21-1 and SCC-Ag assays

Serum CEA and CYFRA21-1 concentrations were measured with Enzymun-TEST CEA and Enzymun-TEST CYFRA21-1 (Boehringer Mannheim, Mannheim, Germany), respectively. SCC-Ag levels were measured in serum with an immunoradiometric Assay kit (Dynabott, Tokyo, Japan). The cutoff values for serum CEA, CYFRA21-1, and SCC-Ag levels were 4.6 ng/ml, 2.57 ng/ml and 1.5 ng/ml, respectively, in accordance with the manufacturer's instructions. The specificity at these cut-off values is 95%.

### Immunological screening of the esophageal carcinoma cell antigens by SEREX

Esophageal carcinoma antigens were screened by SEREX as previously published by Sahin *et al*. [8]. *Escherichia coli XL1*-Blue MRF' was infected with λZAP II phage which contained a cDNA library, and the expression of cDNA was induced by blotting on nitrocellulose membranes (NitroBind, Osmonics Inc., Minnetonka, MN), which had been pretreated with 10 mM isopropyl β-D-thiogalactoside (IPTG, Wako, Kyoto, Japan) for 30 min. The membranes were then washed three times with TBS-T [20 mM Tris-HCl (pH 7.5), 0.15 M NaCl and 0.05% Tween-20], and blocking was performed by treatment with 1% protease-free bovine serum albumin (Wako) in TBS-T for 1 h. The membranes were exposed in 1:2000-diluted serum for 1 h. After washing with TBS-T three times, the membranes were treated with 1:5000-diluted alkaline phosphatase-conjugated Fc fragment-specific goat anti-human IgG and making it react for 1 h. Positive reactions were detected by incubation in color development solution [100 mM Tris-HCl (pH 9.5), 100 mM NaCl and 5 mM MgCl_2_] containing 0.3 mg/ml of nitroblue tetrazolium chloride (Wako) and 0.15 mg/ml of 5-bromo-4-chloro-3-indolyl-phosphate (Wako). Positive clones were recloned twice to obtain monoclonality and retested for serum reactivity.

### Sequence analysis of identified antigens

Monoclonalized phage cDNA clones were converted to pBluescript phagemid by *in vivo *excision using the ExAssist helper phage (Stratagene, La Jolla, CA). Plasmid DNA was obtained from *Escherichia coli *SOLR strain transformed by the phagemid. The cDNA inserts were sequenced by the dideoxy chain termination method using the DNA Sequencing kit BigDyeTM Terminator (Applied Biosystems, Foster City, CA) and ABI PRISM 3700 DNA Analyzer (Applied Biosystems). Sequences were analyzed for homology with public databases of known genes and proteins using NCBI-BLAST.

### Western blotting analysis

*Escherichia coli *JM109 cells which contained cDNA clones recombined in pBluescript II were cultured with or without 0.1 mM IPTG for 2.5 h. Cells were then washed with phsphate-buffered saline and lysed by incubation at 100°C for 3 min in SDS sample buffer [26]. *Escherichia coli *lysate was then subjected to SDS-PAGE followed by western blotting using sera of 73 patients with esophageal cancer, 14 with breast cancer, 9 with gastric cancer, and 16 with colorectal cancer.

### Statistical analyses

The chi-square test or Fisher exact test (2-sided) was performed. The survival probabilities were calculated using the product-limit method of Kaplan and Meier, considering all deaths. Survival differences between groups were determined using the log-rank test. Fisher's exact probability test was used to determine the significance of the differences between two groups. All statistical analyses were carried out using the Stat View 5.0 program for Windows (SAS Institute Inc., Cary, NC). P values at the 0.05 level were considered statistically significant.

### Reverse transcription-PCR (RT-PCR)

Total cellular RNA was isolated from the T.Tn esophageal SCC cell line using AquaPure RNA Isolation Kit (Bio-Rad, Hercules, CA), or from the tumor tissues using FastPure RNA Kit (Takara Bio, Otsu, Japan). RNA of normal esophageal keratinocytes was purchased from Cybrdi (Frederick, MD). Reverse transcription was performed with an oligo(dT)_20 _primer using the ThermoScript RT-PCR System (Invitrogen). The presence of MKRN1 transcripts was examined via PCR amplification using the following primers: sense, 5'-AACGGGTGCCTCAGTGTCCTT-3'; antisense, 5'-CTCCTGATGTTGTGGCTGTTG-3'. β-actin-specific PCR primers (sense, 5'-ACCACAGCTGAGAGGGAAATC-3'; antisense, 5'-AGCACTGTGTTGGCATAGAGG-3') were used as a loading control. PCR was performed using KOD-Plus-DNA polymerase (Toyobo, Osaka, Japan) as follows: an initial denaturation step at 94°C for 3 min, followed by 33 cycles for MKRN1 or 25 cycles for β-actin of denaturation at 94°C for 15 sec, annealing at 60°C for 30 sec, and extension at 68°C for 30 sec, with a final extension of 5 min at 68°C.

### Isolation of stable transfectants

Activated Ha-*ras*-transformed NIH3T3 mouse fibroblasts (clone F25) (ras-NIH3T3) [27,28] were cultured in Dulbecco's modified Eagle's medium (Nissui Pharmaceutical, Tokyo, Japan) supplemented with 5% heat-inactivated bovine serum. Eukaryotic expression vector pME18S-FL3 containing human MKRN1 cDNA was purchased from Toyobo (Osaka, Japan). ras-NIH3T3 cells (5 × 10^4 ^cells) were co-transfected with MKRN1 cDNA and *neo *gene using Lipofectamine reagent (Invitrogen) as described [28,29]. After culture in the presence of G418 (400 μg/ml) for two weeks, surviving colonies were isolated.

### Peptide synthesis and generation of polyclonal antibodies

Two peptides consisting of amino acids between 104 and 118 (CRYEHSKPLKQEEAT) and of amino acids between 399 and 412 (EPQRQKVGTSSRYR) from the MKRN1 protein were synthesized and called MKRN1-K1 and K2, respectively. The peptide was coupled to keyhole limpet haemocyamine that had been activated with N-succinimidyl-3-maleinimido-proprionate. To obtain antibodies, rabbits (bastards) were immunized by subcutaneous injections of 300 μg of coupled peptide emulsified in Freund's complete adjuvant. The immunization was repeated twice at 2-week intervals with the same amount of coupled peptide in Freund's incomplete adjuvant. The titer was tested after each bleed by enzyme-linked immunosorbent assay (ELISA).

Peptide-specific antibodies were enriched by using the pretested rabbit serum columns (Amersham Biosciences, Piscataway, NJ) according to the manufacturer's instructions. In brief, 6 mg of the MKRN1 peptide were coupled to the NHS-activated Sepharose column. Six ml of the rabbit serum were diluted 1:10 with 10 mM Tris-HCl (pH 7.5) and the diluted serum was applied twice to the equilibrated column. Antibodies were eluted with 100 mM glycine buffer (pH 2.5). Fractions were immediately neutralized with 1 M Tris-HCl (pH 8.0). The final concentration of the purified serum was 1:1.83.

### Peptide mass fingerprinting

Extracts of MKRN1-transfected ras-NIH3T3 cells were immunoprecipitated with anti-ubiquitin antibody (Santa Cruz) followed by SDS-polyacrylamide gel electrophoresis as described previously [30]. The gel was stained with silver (Invitrogen) and the gel pieces containing 80- and 82-kDa proteins were washed with water and dehydrated with acetonitrile. After removing acetonitrile, the gel piece was dried in a vacuum centrifuge, supplied with 10 mM dithiothreitol in 100 mM ammonium bicarbonate, and incubated for 1 h at 56°C. The solution was replaced by the same volume of 55 mM iodoacetamide in 100 mM ammonium bicarbonate and incubated for 45 min at room temperature in the dark. The gel piece was washed with 100 mM ammonium bicarbonate, dehydrated with acetonitrile, rehydrated with 100 mM ammonium bicarbonate, dehydrated again with acetonitrile, and dried in a vacuum centrifuge following elimination of acetonitrile. The dried gel piece was immersed in trypsin solution containing 10 μg/ml trypsin in 50 mM ammonium bicarbonate and 5 mM calcium chloride, incubated for 45 min at 4°C, and further at 37°C for 12 h. Peptides were extracted using sequential steps of 20 mM ammonium bicarbonate, followed by 5% formic acid in 50% acetonitrile. The extract was dried in a vacuum centrifuge, supplied with 0.1% trifluoroacetic acid and desalted using ZipTip C18 (Millipore) according to the manufacturer's protocol. The peptides were eluted with 0.1% trifluoroacetic acid in 50% acetonitrile and applied onto MALDI plate.

### Mass spectrometry

Mass spectrometry of peptides was performed using MALDI-TOF MS AXIMA-CFR (Shimadzu Corporation, Tsukuba, Japan). Protein identification was performed using Mascot search (Matrix Science, http://www.matrixscience.com/).

## Results

### Serological screening of T.Tn cell cDNA library

A total of approximately 2 × 10^7 ^pfu from the T.Tn cDNA library were screened using serum samples derived from 21 patients with esophageal SCC. One of the isolated clones, 15J1-3, showed strong homology with the makorin1 (MKRN1; NCBI accession number: NM_013446) gene. MKRN1 appeared to be of much interest because its expression is much higher in the brain and testis than other tissues [31]. A recent report revealed that MKRN1 is involved in the differentiation of various tissues including osteoblasts, eye epithelial cells and kidney [32].

### Immune recognition of MKRN1 gene product in patients with eophageal SCC

**Figure 1:**
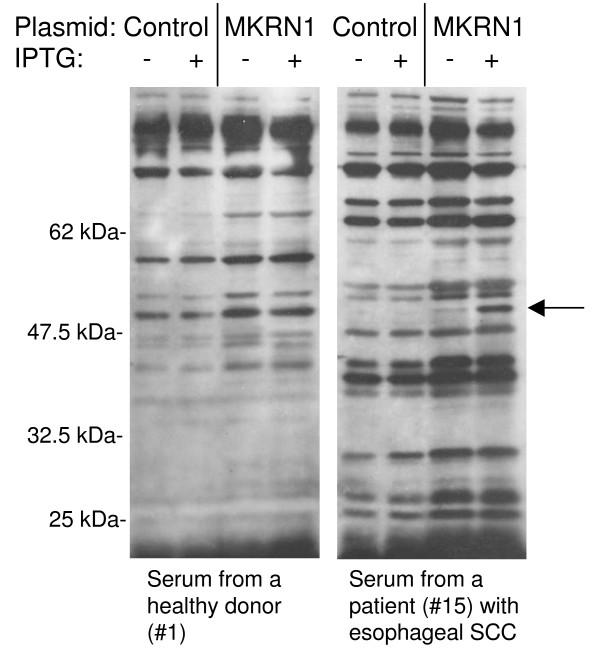
**Recognition of MKRN1 by serum antibodies in patients with esophageal SCC**. *Escherichia coli *containing MKRN1 cDNA expression plasmid (MKRN1) or control empty plasmid (Control) was treated with (+) or without (-) IPTG for 2.5 h, and cell lysates were subsequently subjected to Western blot analysis using the sera from healthy donor #1 and esophageal SCC patients #15 as indicated. An arrow indicates the IPTG-induced polypeptide that represents the encoded MKRN1 cDNA product.

The existence of antibodies against the MKRN1 gene product was examined with 73 sera of patients with esophageal SCC and 43 sera of healthy donors by Western blot analysis. In order to confirm that the antigen reacting with serum antibodies derives from the insert cDNA, protein extracts of IPTG-treated *Escherichia coli *bearing MKRN1 cDNA expression plasmids or the control empty plasmids were compared with those of non-treated bacteria. The 50-kDa band which appeared only in MKRN1-expressing bacteria in response to IPTG was judged as positive. Figure 1 shows the representative results of s-MKRN1-Abs-negative serum of a healthy donor, #1, and s-MKRN1-Abs-positive serum of a patient, #15. For this 50-kDa band, 18 of 73 (25%) sera of patients with esophageal SCC were sero-positive (data not shown). In contrast, all 43 healthy donors were sero-negative. Similarly, positive bands were detected in the sera of two of 14 (14%) breast cancer patients, none of the 9 (0%) gastric cancer patients and six of the 16 (38%) colon cancer patients (data not shown).

### Presence of serum anti-MKRN1 antibodies and clinicopathological variables of esophageal SCC

**Figure 2:**
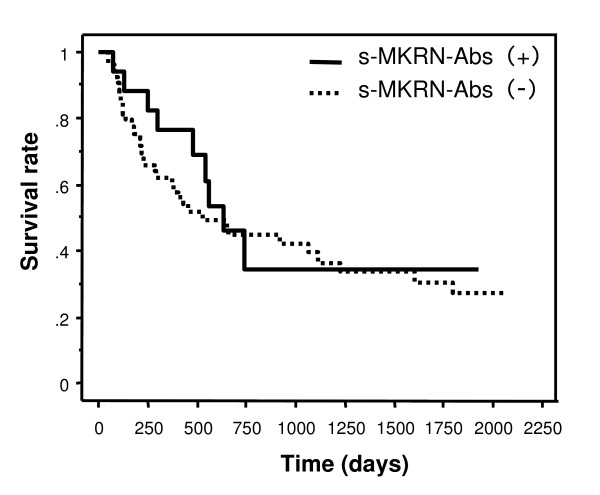
**Kaplan-Meier overall survival curves for the patients with or without s-MKRN1-Abs**. Logrank (Mantel-Cox) *P*-value = 0.5209.

We then examined the relationship between the presence of s-MKRN1-Abs and the clinicopathological features of the patients (Table 1). As for histopathological grading, well-differentiated tumors (G1) were associated with the presence of s-MKRN1-Abs significantly compared to poorly differentiated tumors (G3), and vice versa (*P *= 0.008). Nodal involvement was more frequently observed in the patients with s-MKRN1-Abs than in those without s-MKRN1-Abs, yet the difference was not statistically significant (*P *= 0.194). There was no correlation between the presence of s-MKRN1-Abs and other features such as age, gender, location, tumor sizes, depth of tumor, distant metastasis and stages. Kaplan-Meier plotting showed no significant correlation between s- MKRN1-Abs and survival (Figure 2).

### Correlation between the presence of s-MKRN1-Abs and positivity of conventional serum tumor markers

When the patients were divided into two groups according to the presence of s-MKRN1-Abs, there was no significant correlation between the presence of s-MKRN1-Abs and the positivity of conventional serum tumor markers (Table 1). Consequently, the sensitivity of serum markers to detect esophageal SCC was improved by using s-MKRN1-Abs in combination with conventional markers. For example, the positivity increased from 36% of SCC-Ag alone to 51 % by combination of s-MKRN1-Abs and SCC-Ag.

### Increased expression of MKRN1 mRNA in esophageal SCC

**Figure 3:**
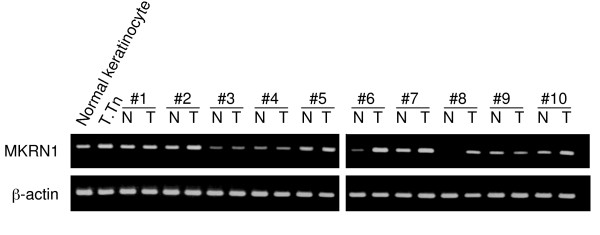
**Elevated levels of MKRN1 mRNA in esophageal SCC tissues**. MKRN1 (upper panel) and β-actin (lower panel) mRNA levels were examined by RT-PCR in specimens of normal (N) and carcinoma (T) tissues resected from patients 1 to 10 (#1 – #10). 'Normal keratinocyte' and 'T.Tn' represent the products from RNA of normal esophageal keratinocytes obtained from Cybrdi and the T.Tn esophageal SCC cell line, respectively.

The expression levels of MKRN1 mRNA in esophageal tissue specimens of SCC or normal cells were examined by RT-PCR analysis. T.Tn esophageal SCC cells, as a positive control, showed higher MKRN1 mRNA levels than normal esophageal keratinocytes (Figure 3). MKRN1 mRNA levels in carcinoma tissues were higher than those in the normal counterparts of patient numbers 2, 6, 7, 8 and 10. Thus, this elevated expression may account for the development of s-MKRN1-Abs.

### Ubiquitination of L-FILIP was stimulated by MKRN1

**Figure 4:**
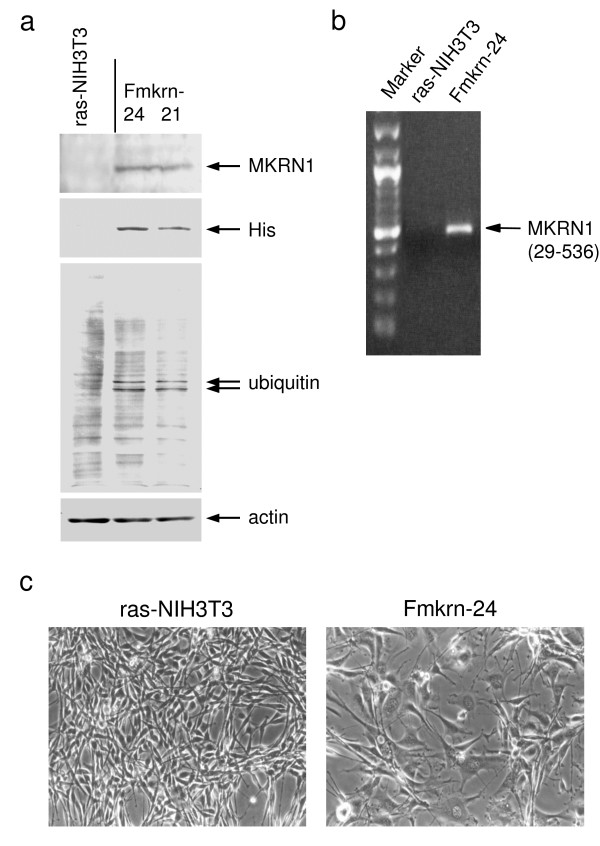
**Isolation of stable MKRN1 transfectants**. (a) Protein expression levels of MKRN1, His-tagged proteins, ubiquitinated proteins and β-actin in MKRN1 transfectants, Fmkrn-24 and 21, and the parental ras-NIH3T3 cells were examined by Western blotting using the anti-MKRN1 peptide antibody, the anti-His antibody (lower panel), the anti-ubiquitin antibody or the anti-β-actin antibody. (b) Expression of MKRN1 mRNA in MKRN1 transfectant Fmkrn-24 and ras-NIH3T3 cells was examined by RT-PCR. MKRN1 sequence between nucleotide positions 29 and 536 was amplified. The position of PCR products is indicated by an arrow. (c) Phase morphology of ras-NIH3T3 and Fmkrn-24 cells. Original magnification, ×100.

**Figure 5:**
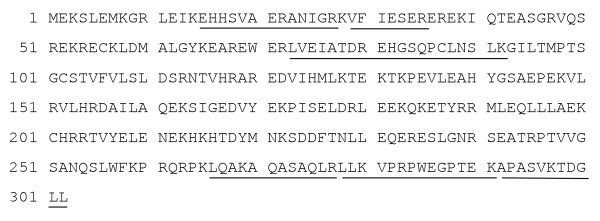
**Identification of L-FILIP as MKRN1-induced ubiquitinated proteins**. Ubiquitinated proteins of 80 and 82 kDa in Fmkrn-24 cells were immunoprecipitated with anti-ubiquitin antibody followed by SDS-polyacrylamide gel electrophoresis. The gel pieces containing the 80- and 82-kDa proteins were excised, and the tryptic peptides were analyzed by mass spectrometry. Amino acid sequences of L-FILIP are shown. The identical amino acids sequences between the analyzed peptides and L-FILIP are shown by underlines.

To examine the biological role of MKRN1, human MKRN1 cDNA was recombined in pcDNA3-Myc, His and transfected into ras-NIH3T3 cells as described [28]. The protein (Figure 4a) and mRNA (Figure 4b) of transduced MKRN1 were observed in a stable transfected clone, Fmkrn-24, but not in the parental ras-NIH3T3 cells. The phase morphology of Fmkrn-24 was markedly different from the parental cells; i.e. enlargement of cell bodies with flat and extended cell periphery (Figure 4c). Because E3 ubiquitin-protein ligase activity was suspected to MKRN1 [31,33], ubiquitinated proteins were examined by Western blotting using anti-ubiquitin antibody. Two ubiquitinated proteins of 80 and 82 kDa were clearly observed in two MKRN1 transfectants but not in the parental ras-NIH3T3 cells (Figure 4a). These two proteins in Fmkrn-24 cells were immunoprecipitated with anti-ubiquitin antibody, separated by SDS-polyacrylamide gel electrophoresis and analyzed by mass spectrometry. Six tryptic polypeptides were completely matched to the sequence of mouse L-FILIP (filamin A interacting protein 1) (Figure 5). This suggests that the ubiquitination of L-FILIP was induced by MKRN1.

## Discussion

In the current study, we applied SEREX analysis to esophageal SCC, and identified MKRN1. MKRN1 locus maps to a conserved syntenic group near the T-cell receptor cluster in chromosome 7q34 [31]. MKRN1 is widely expressed in mammals at high levels in the murine embryonic nervous system and adult testis. Thus far, several MKRN1-related genes have been reported, and MKRN1 is the ancestral founder of the MKRN1 gene family. This family encodes putative ribonucleoproteins with a distinct collection of four zinc-finger motifs followed by a RING zinc-finger [31]. E3 ubiquitin-protein ligase activity is intrinsic to RING domain-containing molecules such as Mdm2 and c-Cbl. Kim *et al*. have shown that MKRN1 binds and promotes ubiquitination of TERT and subsequent proteasomal degradation [33]. On the other hand, Hirotsune *et al*. have reported that a putative pseudogene-induced destabilization of MKRN1 led to defects of differentiation such as polycystic kidneys and bone deformity [32].

The presence of serum MKRN1 antibodies (s-MKRN1-Abs) in cancer patients was examined by Western blotting (Figure 1). s-MKRN1-Abs were frequently observed in patients with esophageal SCC (25%) and colon carcinoma (38%) but not in healthy donors. Even in patients with early-stage (T1) esophageal tumors, s-MKRN1-Abs were positive for 3 (16%) of 19 patients (Table 1). Thus, s-MKRN1-Abs can be a candidate of diagnostic markers of esophageal SCC with low false positive rates.

There was no correlation between the presence of s-MKRN1-Abs and clinicopathological variables except histological grading (Table 1). Well-differentiated type tumors were observed more frequently in the patients with s-MKRN1-Abs than in the patients without s-MKRN1-Abs. This is consistent with the report of Hirotsune *et al*. that MKRN1 has a crucial role in differentiation [32].

RT-PCR analysis showed that the expression of MKRN1 mRNA was frequently higher in esophageal SCC tissues than in the peripheral normal esophageal mucosa (Figure 3). Densitometric analysis revealed that the relative expression levels of MKRN1 versus β-actin mRNA in esophageal SCC were approximately 2.5-fold higher than in the normal esophageal mucosa. This elevated expression may, in part, account for the development of serum antibodies, and give a rationale of application of s-MKRN1-Abs as a tumor marker.

Stable transfection of ras-NIH3T3 cells with MKRN1 cDNA showed prominent morphological changes (Figure 4), which resembled phenotypic reversion of ras-NIH3T3 cells to NIH3T3-like cells [34]. This suggests an effect of MKRN1 on actin cytoskeleton because such changes were frequently associated with reorganization of actin stress fibers [35]. In addition, ubiquitination of 80- and 82-kDa proteins was induced by transfection with two MKRN1-transfected cells (Figure 4a). Mass spectrometry revealed that these ubiquitinated proteins are L-FILIP (Figure 5). FILIP interacts with filamin A, a component of stress fibers, and induces degradation of filamin A, leading to alteration of cell shape and decrease in cell motility [36,37]. Therefore, MKRN1-induced ubiquitination of L-FILIP followed by degradation with proteasome system may explain the prominent morphological changes in MKRN1-transfected cells as well as the effects on differentiation.

The expression of MKRN1 is increased in half (5 out of 10 cases) but not all esophageal SCC tissues (Figure 3). Therefore, it is implausible that MKRN1 has an indispensable role in esophageal carcinogenesis. However, involvement in differentiation and the effects on the cytoskeleton via L-FILIP suggest that MKRN1 has a key role in a certain type of esophageal carcinogenesis. Suppression of MKRN1 activity might be a novel strategy for esophageal SCC therapy.

## Conclusion

It is plausible that MKRN1 is involved in differentiation and carcinogenesis of a certain type of esophageal SCC. s-MKRN1-Abs can be a candidate of diagnostic markers of esophageal SCC.

## Abbreviations

IPTG: isopropyl β-D-thiogalactoside; ORF: open reading frame; RT-PCR: reverse transcription-PCR; SCC: squamous cell carcinoma; SEREX: serological identification of antigens by recombinant cDNA expression cloning; s-MKRN1-Abs: serum anti-MKRN1 antibodies.

## Competing interests

The authors declare that they have no competing interests.

## Authors' contributions

HS carried out gene manipulation and the statistical analysis. TS carried out the immunoassays. MY carried out expression cloning. AK carried out gene expression analysis. MK carried out transfection experiments. FN and MT participated in coordination. TO and HM contributed in the clinicopathological analysis. TH participated in the design of the study. All authors read and approved the final manuscript.

## Pre-publication history

The pre-publication history for this paper can be accessed here:

http://www.biomedcentral.com/1471-2407/9/232/prepub
